# Insulin-like growth factor-1 levels are associated with interventricular septal thickening

**DOI:** 10.3389/fendo.2022.997023

**Published:** 2022-12-07

**Authors:** Yuan Chen, Xinchun Cheng, Suli Li, Yuting Yin, Shuqing Xing, Yanying Guo

**Affiliations:** ^1^ Graduate School of Xinjiang Medical University, Urumqi, China; ^2^ Geriatrics Center, People’s Hospital of Xinjiang Uygur Autonomous Region, Urumqi, China; ^3^ Department of Endocrinology and Metabolic Diseases, People’s Hospital of Xinjiang Uygur Autonomous Region, Xinjiang Clinical Research Center for Diabetes, Urumqi, China

**Keywords:** insulin-like growth factor-1, interventricular septal thickening, linear association, case–control study, blood pressure

## Abstract

**Background and objective:**

Insulin-like growth factor-1 (IGF-1) regulates cardiomyocyte survival, hypertrophy, and ageing. We aimed to investigate the potential correlation between IGF-1 and interventricular septal (IVS) thickening.

**Methods:**

Medical record data were obtained from patients hospitalized between May 1, 2012 and April 30, 2022. All patients underwent echocardiography and had laboratory data on plasma IGF-1. We analyzed the relationship between IGF-1 levels and IVS thickening based on logistic regression models.

**Results:**

Propensity score matching at 1:4 ratio was performed for 180 patients with IVS thickening and 1,964 patients without IVS thickening. Finally, 180 (case group) and 623 (control group) patients were enrolled. Of the total 803 patients, the mean age was 59 years, and 59.7% were male. In multivariate-adjusted models that adjusted for propensity scores, the risk of IVS thickening increased with increasing IGF-1 levels; specifically, the risk of IVS thickening increased per 1 ng/mL [odds ratio (OR) 1.003; 95% confidence interval (CI): 1.002–1.004; *P* < 0.001], per 5 ng/mL (OR, 1.016; 95% CI, 1.010–1.022; *P* < 0.001), and per 10 ng/mL(OR, 1.032; 95% CI, 1.019–1.045; *P* < 0.001) increase in IGF-1 levels. When the IGF-1 levels were expressed as a categorical variable, the increased levels of IGF-1 led to an increased risk of IVS thickening; specifically, the OR of IVS thickening for T3 >152.00 ng/mL was 2.020 (95% CI: 1.310–3.115, *P* < 0.01) compared with T1 <102.00 ng/mL. We performed restricted cubic splines, and it showed a linear association between IGF-1 levels and the risk of IVS thickening. In splines for the age and sex subgroups, different IGF-1 levels increased the risk of IVS thickening among different age groups in male patients: 18–44 years when IGF-1 value >164.00 ng/mL, 45–60 years when IGF-1 value > 140.34 ng/mL and ≥ 60 years when IGF-1 value >108.20 ng/mL. In female patients aged 45–60 years, the risk of IVS thickening increased when the IGF-1 levels were >207.45 ng/mL. However, IGF-1 was not significantly correlated with IVS thickening in female patients aged 18–45 and ≥60 years. Sensitivity analysis by excluding those with acromegaly did not change the relationship between IGF-1 and the risk of IVS thickening.

**Conclusion:**

The plasma IGF-1 levels were related to the risk of IVS thickening irrespective of blood pressure.

## Introduction

Cardiomyopathy is either caused by the heart muscle itself or as a side effect of some other systemic diseases, resulting in heart damage and electrical dysfunction. Cardiomyopathies are classified into two major groups, primary and secondary, according to the American Heart Association (1, 2). Interventricular septal (IVS) thickening is one of the clinical manifestations of cardiomyopathies. IVS is detected by echocardiography and caused by hypertension and hypertrophic cardiomyopathy among others. Left ventricular hypertrophy and IVS thickness are important risk factors for cardiovascular mortality and all-cause mortality (3, 4). IVS thickening is suggested to be a type or an early phase of hypertensive heart disease, even in the presence of normal left ventricular mass (5, 6). Isolated IVS thickening defined by twice the thickness of the septum divided by the left ventricular internal diameter >0.45, although rare, may be present in the general population (7). Moreover, higher fasting blood glucose (FBG) and uric acid are associated with IVS thickening among non-diabetic patients who are overweight and obese (8). Therefore, it is vital to analyze other risk factors that cause IVS thickening in addition to hypertension.

Insulin-like growth factor-1 (IGF-1) is a major regulator of postnatal somatic growth, mediating many effects of growth hormone (GH). IGF-1 is mainly synthesized by the liver and binds to its ubiquitously expressed cognate receptor, IGF-1 receptor (IGF-1R) (9, 10). IGF-1 directly affects the contractility of cardiomyocytes and increases the intracellular calcium concentration and calcium sensitivity in the myofilaments located within cardiomyocytes (11, 12). Animal studies of cardiomyocyte-specific knockout or transgenic IGF-1R mice showed that IGF-1R regulates cardiomyocyte survival, hypertrophy, and ageing by activating the phosphoinositide 3-kinase (PI3K)/Akt signaling pathway through its intrinsic tyrosine kinase activity (13–15). Therefore, it is necessary to evaluate the relationship between IGF-1 and IVS thickening.

Considering these data, our case–control study was designed to identify the potential relationship between IGF-1 levels and IVS thickening and discuss the risk level according to different age and sex groups.

## Materials and methods

### Study population

Medical record data were obtained from patients hospitalized in the People’s Hospital of Xinjiang Uygur Autonomous Region from May 1, 2012 to April 30, 2022.

**Figure 1:**
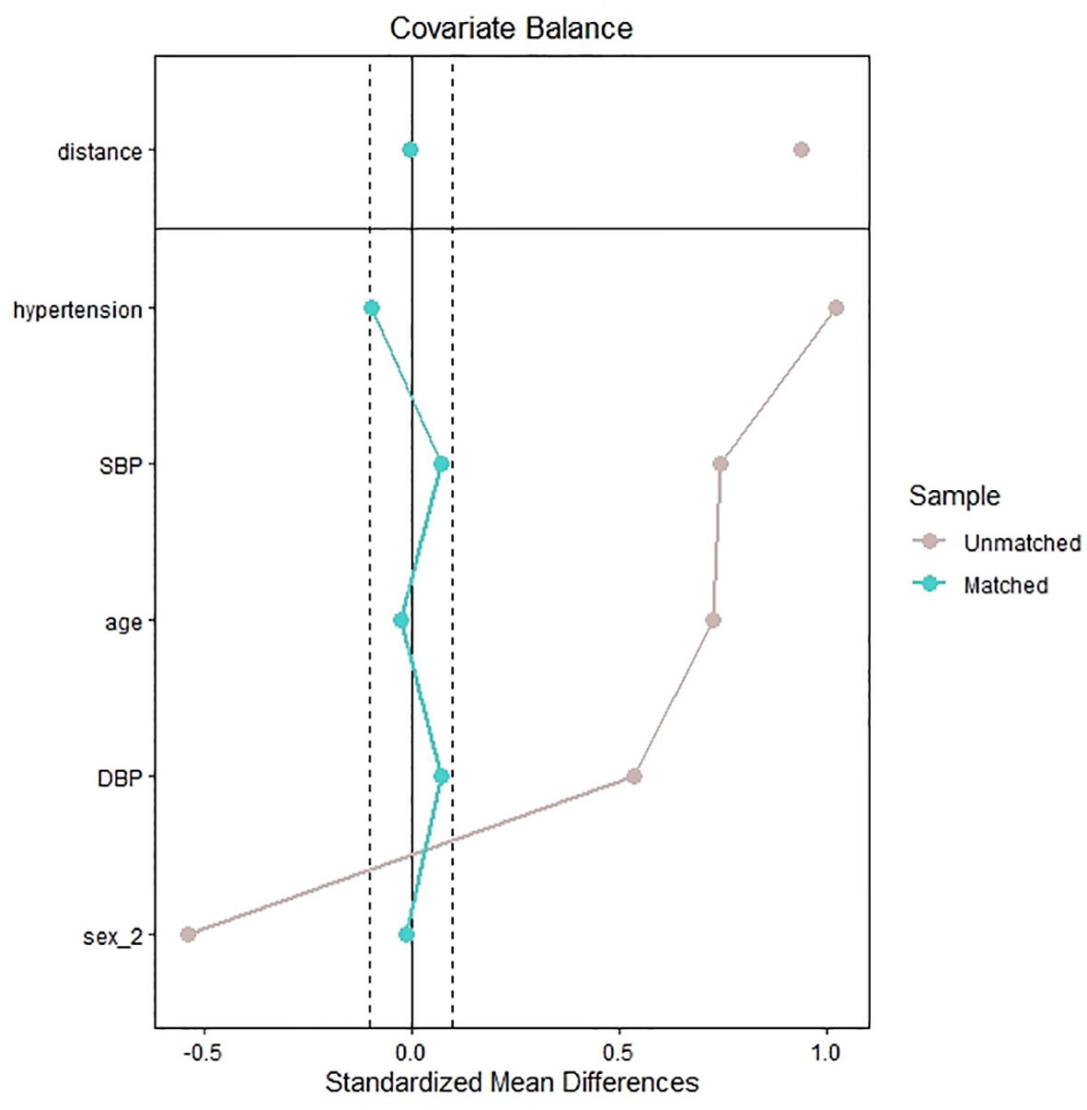
Standardized mean difference of the matching covariates between the case group and control group before and after propensity score matching.

The hospital has 2,700 beds and treats nearly 160,000 patients per year. We used the internal database search engine to extract electronic medical record data. The patients with IVS thickening (case group) were matched with the patients without IVS thickening (control group) (1:4 ratio) by age, sex, history of hypertension, and levels of systolic blood pressure (SBP) and diastolic blood pressure (DBP) (**Figure 1**). The Ethics Committee of the People’s Hospital of Xinjiang Uygur Autonomous Region approved the study.

The inclusion criteria were as follows:

(1) patients who had echocardiography performed during the hospital stay and
(2) patients who had laboratory data on plasma IGF-1 levels.

The diagnosis and screening of acromegaly followed the Endocrine Society Clinical Practice Guidelines (16).

### Clinical data collection and laboratory measurements

Based on the electronic medical record system, we collected all the clinical data at admission, including the age, sex, body mass index (BMI), smoking or alcohol consumption status (never, former/current), history of coronary artery disease (CAD), hypertension and diabetes mellitus (DM), levels of SBP and DBP, heart rate, and medical diagnoses. We obtained the patients’ use of the following medications before admission: angiotensin-converting enzyme inhibitor (ACEI)/angiotensin-2 receptor blocker (ARB), beta-blockers (β blockers), calcium channel blockers, diuretics statins and antiplatelet agents. We also obtained the following laboratory data from the laboratory information system: alanine transaminase (ALT), aspartate transaminase (AST), γ-gamma-glutamyltransferase (γ-GGT), alkaline phosphatase (ALP), albumin, creatinine (Cr), blood urea nitrogen (BUN), uric acid (UA), triglyceride (TG), total cholesterol (TC), low-density lipoprotein cholesterol (LDL-C), high-density lipoprotein cholesterol (HDL-C), serum calcium, serum potassium, serum natrium, serum phosphate, FBG, creatine kinase (CK), creatine kinase isoenzyme MB (CK-MB), white blood cell (WBC), platelet (PLT), hemoglobin (Hb), D-dimer, cortisol, adrenocorticotropic hormone (ACTH), thyroid-stimulating hormone (TSH), free triiodothyronine (FT3), free thyroxine (FT4), total triiodothyronine (TT3), total thyroxine (TT4), IGF-1, and GH.

All blood samples were collected from fasting blood. Biochemical indicators were measured by enzymatic methods using an autoanalyzer (7600-010 Automatic Analyzer: Hitachi Medical Systems, Suzhou, China). IGF-1 and GH were detected by immunochemiluminescence method, using DPC Immulite 2000 chemiluminescence instrument (Siemens AG, Munich, Germany).

### Assessment of IVS thickness

An experienced sonographer performed the echocardiography on all patients. The data for conclusion echocardiography were collected from our hospital’s Picture Archiving and Communication System. The diagnosis of IVS thickening was based on echocardiography with an interventricular septal thickness >11 mm (17).

### Statistical analysis

We used R language (version 4.1.0) for data analysis. Normally distributed variables are presented as mean ± standard deviation and were compared by using Student’s *t*-tests. Non-normally distributed variables are presented as median ± interquartile range and were compared by using the Mann–Whitney *U*-test. Categorical variables are expressed as frequencies and proportions and were compared with the chi-square test. We analyzed the IGF-1 levels as a continuous variable. Then, the study participants were classified based on tertiles of IGF-1 levels (T1: 102.00, T2: 102.00–152.00, and T3 > 152.00 ng/mL). Propensity scoring and matching were conducted using the “MatchIt” package with a nearest neighbor matching algorithm and a caliper of 0.2. We performed collinearity statistics by using multivariable linear regression analysis among all variables. Multicollinearity was judged if the tolerance was <0.2 and the variance inflation factor was >5, and AST, TC, and TT3 were removed before the multivariable analysis (**Supplementary Table 1**). The propensity scores were calculated for all potential confounding variables shown in **Table 1** (eliminating AST, TC, and TT3). To evaluate the association between IGF-1 and IVS thickening, we performed Univariate model, model 1 (adjusting for age, sex, BMI, levels of SBP and DBP, history of hypertension, and DM and CAD) and model 2 (adjusting for propensity scores)—based on logistic regression models. Furthermore, we used restricted cubic splines with three knots at the 10th, 50th, and 90th centiles to flexibly model the association between IGF-1 levels and IVS thickening based on model 2. Finally, an adjusted propensity score model was used for the analysis of prespecified subgroups, defined according to age (<45 or ≥45 years), sex (male or female), BMI (<28 or ≥28 kg/m^2^), hypertension (yes or no), DM (yes or no), CAD (yes or no), acromegaly (yes or no), and use of ACEI/ARB agents. Subgroup analyses were done to detect the presence of any interaction between IGF-1, as a continuous and categorical variable, and the risk of IVS thickening. We also conducted a sensitivity analysis, which excluded patients with acromegaly, to evaluate the relationship between IGF-1 levels and the risk of IVS thickening in patients without acromegaly. *P <*0.05 was considered statistically significant.

**Table 1:** Baseline characteristics of the case group and control group.

Variables	Control group *N* = 623	Case group *N* = 180	*P*-value
Age (years)	58.0 (49.0; 68.0]	59.0 (51.0; 69.2]	0.314
Sex (*n*, %)			0.161
Male	363 (58.3%)	116 (64.4%)	
Female	260 (41.7%)	64 (35.6%)	
Smoking (*n*, %)			0.351
Never	425 (68.2%)	130 (72.2%)	
Former or current	198 (31.8%)	50 (27.8%)	
Alcohol consumption status (*n*, %)			0.483
Never	470 (75.4%)	141 (78.3%)	
Former or current	153 (24.6%)	39 (21.7%)	
Diabetes mellitus (*n*, %)			1.000
No	318 (51.0%)	92 (51.1%)	
Yes	305 (49.0%)	88 (48.9%)	
CAD (*n*, %)			0.075
No	504 (80.9%)	134 (74.4%)	
Yes	119 (19.1%)	46 (25.6%)	
Hypertension (*n*, %)			0.919
No	134 (21.5%)	40 (22.2%)	
Yes	489 (78.5%)	140 (77.8%)	
SBP (mmHg)	139 (125; 151)	145 (126; 156)	0.047
DBP (mmHg)	83.0 (75.0; 93.0)	84.5 (76.0; 95.0)	0.309
BMI (kg/m^2^)	26.2 (24.4; 29.7)	27.2 (25.4; 31.3)	0.003
Heart rate (beats/min)	80.0 (77.0; 84.0)	80.0 (76.0; 84.0)	0.877
ALT (U/L)	20.0 (14.9; 29.3)	19.0 (14.0; 29.1)	0.604
AST (U/L)	18.0 (15.0; 24.0)	19.0 (14.0; 24.0)	0.980
γ-GGT (U/L)	23.0 (16.1; 38.0)	25.0 (17.8; 39.2)	0.232
ALP (U/L)	70.2 (60.1; 89.7)	74.6 (66.1; 95.2)	0.008
Albumin (g/L)	40.6 (37.2; 44.3)	39.5 (36.3; 42.8)	0.021
Cr (umol/L)	60.9 (51.9; 73.8)	62.4 (49.7; 76.4)	0.645
BUN (mmol/L)	2.31 (0.08; 5.17)	0.16 (0.08; 4.93)	0.562
UA (umol/L)	301 (244; 372)	299 (247; 374)	0.834
TG (mmol/L)	1.35 (1.08; 2.08)	1.34 (1.18; 1.89)	0.744
LDL-C (mmol/L)	2.58 (2.02; 3.14)	2.58 (1.97; 3.11)	0.574
TC (mmol/L)	4.37 (3.81; 5.08)	4.37 (3.55; 4.90)	0.097
HDL-C (mmoL/L)	1.05 (0.90;1.20)	1.01 (0.82;1.09)	0.002
Serum calcium (mmol/L)	2.22 (2.12; 2.31)	2.23 (2.13; 2.32)	0.417
Serum potassium (mmol/L)	3.87 (3.58; 4.14)	3.82 (3.58; 4.07)	0.134
Serum natrium (mmol/L)	140 (138; 142)	141 (138; 142)	0.158
Serum phosphate (mmol/L)	1.16 (1.01; 1.28)	1.17 (1.05; 1.32)	0.113
FBG (mmol/L)	5.08 (4.66; 7.20)	5.09 (4.62; 7.18)	0.834
CK (U/L)	67.0 (52.6; 106)	70.0 (59.6; 102)	0.501
CK-MB (ng/mL)	11.1 (1.52; 16.4)	12.1 (1.87; 16.4)	0.398
WBC (10^9^/L)	6.55 (5.48; 8.09)	6.45 (5.26; 7.96)	0.538
PLT (10^9^/L)	240 (199; 281)	241 (183; 288)	0.727
Hb (g/L)	139 (126; 151)	138 (124; 150)	0.738
D-Dimer (mg/L)	0.38 (0.23; 0.69)	0.43 (0.24; 0.80)	0.193
CortisoL (µg/dL)	8.39 (3.79; 14.3)	8.40 (4.93; 14.0)	0.194
ACTH (pg/mL)	18.6 (13.3; 30.6)	18.6 (12.7; 32.4)	0.953
TSH (µIU/mL)	2.11 (1.19; 3.18)	2.13 (1.09; 2.89)	0.295
FT3 (pg/mL)	2.73 (2.17; 3.02)	2.74 (2.25; 3.04)	0.447
FT4 (ng/dL)	1.16 (1.01; 1.33)	1.17 (1.02; 1.33)	0.381
TT3 (ng/mL)	0.92 (0.72; 1.08)	0.94 (0.74; 1.07)	0.423
TT4 (µg/dL)	6.70 (5.57; 8.02)	6.81 (5.87; 7.82)	0.493
IGF-1 (ng/mL)	122 (87.6; 161)	132 (95.3; 212)	0.001
GH (ug/L)	0.18 (0.09; 0.56)	0.36 (0.12; 1.37)	<0.001
ACEI/ARB (*n*, %)			0.077
No	469 (75.3%)	123 (68.3%)	
Yes	154 (24.7%)	57 (31.7%)	
β blockers (*n*, %)			0.271
No	554 (88.9%)	154 (85.6%)	
Yes	69 (11.1%)	26 (14.4%)	
Calcium channel blockers (*n*, %)			0.354
No	470 (75.4%)	129 (71.7%)	
Yes	153 (24.6%)	51 (28.3%)	
Diuretics (*n*, %)			0.401
No	553 (88.8%)	155 (86.1%)	
Yes	70 (11.2%)	25 (13.9%)	
Antiplatelet agents (*n*, %)			0.788
No	513 (82.3%)	146 (81.1%)	
Yes	110 (17.7%)	34 (18.9%)	
Statin (*n*, %)			0.165
No	537 (86.2%)	147 (81.7%)	
Yes	86 (13.8%)	33 (18.3%)	

SBP, systolic blood pressure; DBP, diastolic blood pressure; BMI, body mass index; CAD, coronary artery disease; DM, diabetes mellitus; ALT, alanine transaminase; AST, aspartate transaminase; γ-GGT, γ-gamma-glutamyltransferase; ALP, alkaline phosphatase; Cr, creatinine; BUN, blood urea nitrogen; UA, uric acid; TG, triglyceride; TC, total cholesterol; LDL-C, low-density lipoprotein cholesterol; HDL-C, high-density lipoprotein cholesterol; FBG, fasting blood glucose; CK, creatine kinase; CK-MB, creatine kinase isoenzyme MB; WBC, white blood cell; PLT, plastocyte; Hb, hemoglobin; ACTH, adrenalotropic hormone; TSH, thyroid-stimulating hormone; FT3, free triiodothyronine; FT4, free thyroxine; TT3, total triiodothyronine; TT4, total thyroxine; IGF-1, insulin-like growth hormone-1; GH, growth hormone ACEI/ARB: angiotensin-converting enzyme inhibitor/angiotensin 2 receptor blocker.

## Results

### Characteristics of the patients

**Figure 2:**
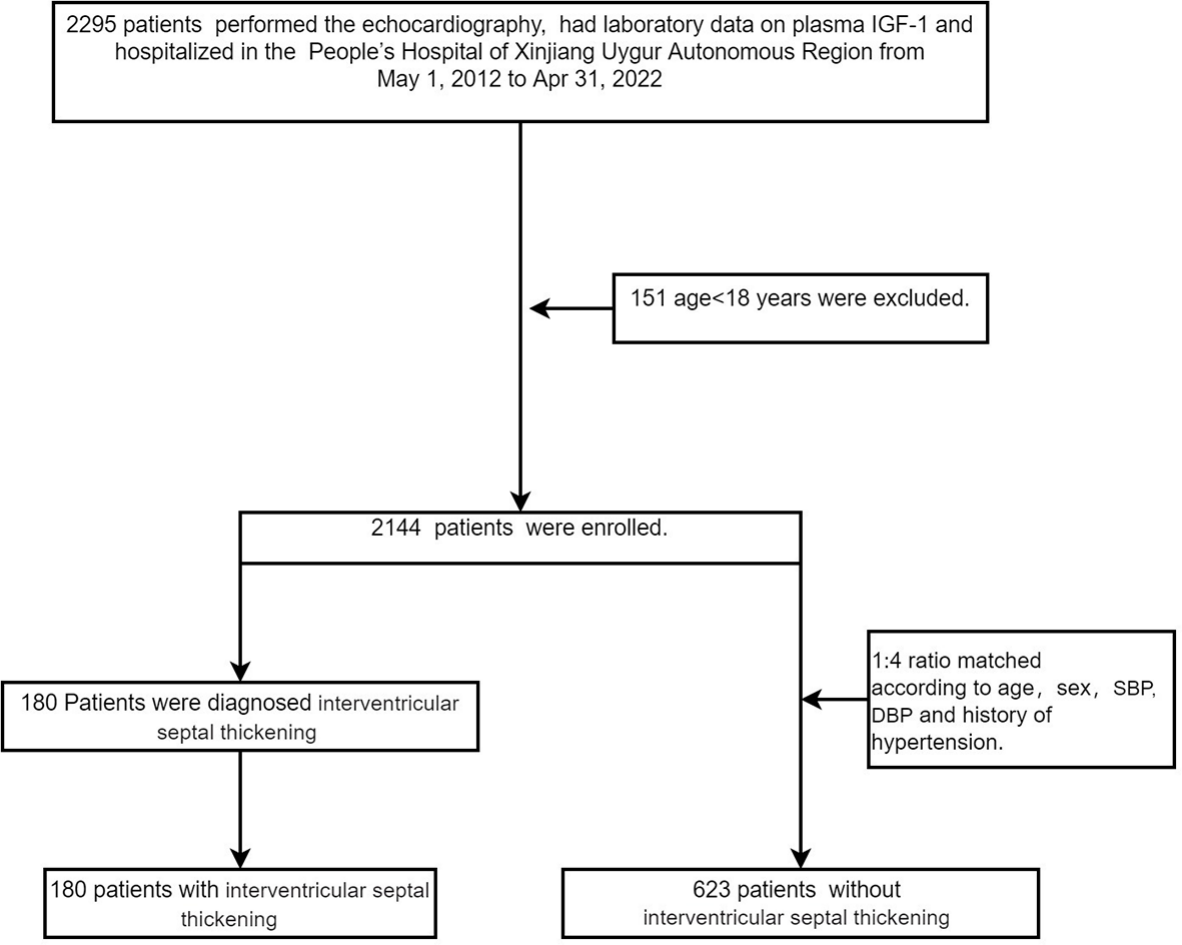
Flow chart of patient selection in our study.

A total of 2,295 patients had laboratory data on plasma IGF-1 and underwent echocardiography. Of the 2,144 eligible patients, 180 patients were diagnosed with IVS thickening (case group) and matched with 623 patients without IVS thickening (control group) (**Figure 2**). **Figure 1** shows the standardized mean difference of the matching covariates between the case group and the control group before and after the propensity score matching. The mean age overall was 59 years, and 59.7% were male. The characteristics of the patients did not differ between the two groups in terms of age, sex, smoking and alcohol consumption status, levels of DBP, history of DM, CAD, and hypertension, heart rate, ALT, AST, γ-GGT, Cr, BUN, UA, TG, LDL-C, TC, serum calcium, serum potassium, serum natrium, serum phosphate, FBG, CK, CK-MB, WBC, PLT, Hb, D-dimer, cortisol, ACTH, TSH, FT3, FT4, TT3, TT4, and use of antihypertensive, antiplatelet, and statin agents. The case group had higher levels of BMI, SBP, ALP, IGF-1, and GH and lower levels of albumin and HDL-C than the control group (**Table 1**).

### Association between IGF-1 and the risk of IVS thickening

The logistic regression analysis showed that the risk of IVS thickening increased per 1 ng/mL increase in IGF-1 (OR, 1.003; 95% CI, 1.002–1.004; *P* < 0.001 in the univariate model; OR, 1.004; 95% CI, 1.002–1.005; *P* < 0.001 in model 1, and OR, 1.003; 95% CI, 1.002–1.004; *P* < 0.001 in model 2). In model 2, per 5 ng/mL (95% CI, 1.010–1.022; *P <*0.001) and per 10 ng/mL (95% CI, 1.019–1.045; *P* < 0.001) increase in IGF-1 showed 1.016- and 1.032-fold increase in odds for the presence of IVS thickening, respectively. When the IGF-1 levels were expressed as a categorical variable, patients in T3 (OR: 2.020, 95% CI: 1.310–3.115, *P* < 0.01) had a markedly higher risk of IVS thickening, while patients in T2 (*P* > 0.05) showed no statistical significance compared with T1 in model 2 (*P* for trend <0.01). A similar pattern was observed in the univariate model and model 1 (**Table 2**).

**Table 2:** The logistic regression analysis shows the relationship between insulin-like growth factor-1 and the risk of interventricular septal thickening.

	Univariate model			Model 1			Model 2		
	OR	95% CI	*P*-value	OR	95% CI	*P*-value	OR	95% CI	*P-*value
IGF-1 per 1 ng/mL	1.003	1.002–1.004	<0.001	1.004	1.002–1.005	<0.001	1.003	1.002–1.004	<0.001
IGF-1 per 5 ng/mL	1.016	1.010–1.022	<0.001	1.018	1.012–1.024	<0.001	1.016	1.010–1.022	<0.001
IGF-1 per 10 ng/mL	1.032	1.021–1.045	<0.001	1.036	1.023–1.049	<0.001	1.032	1.019–1.045	<0.001
IGF group									
T1 <102.00 ng/mL	Ref.								
T2 102.00–152.00 ng/mL	1.060	0.691–1.627	0.788	1.127	0.724–1.754	0.595	1.207	0.768–1.898	0.414
T3 >152.00 ng/mL	1.756	1.169–2.638	0.007	1.968	1.266–3.060	0.003	2.020	1.310–3.115	0.001
*P* for trend			0.006			0.002			0.001

Mode 1: adjusted for age, sex, BMI, DBP, SBP, hypertension, T2DM, and CAD. Model 2: adjusted for propensity scores.

IGF-1, insulin-like growth hormone-1; IVS thickening, interventricular septal thickening.

**Figure 3:**
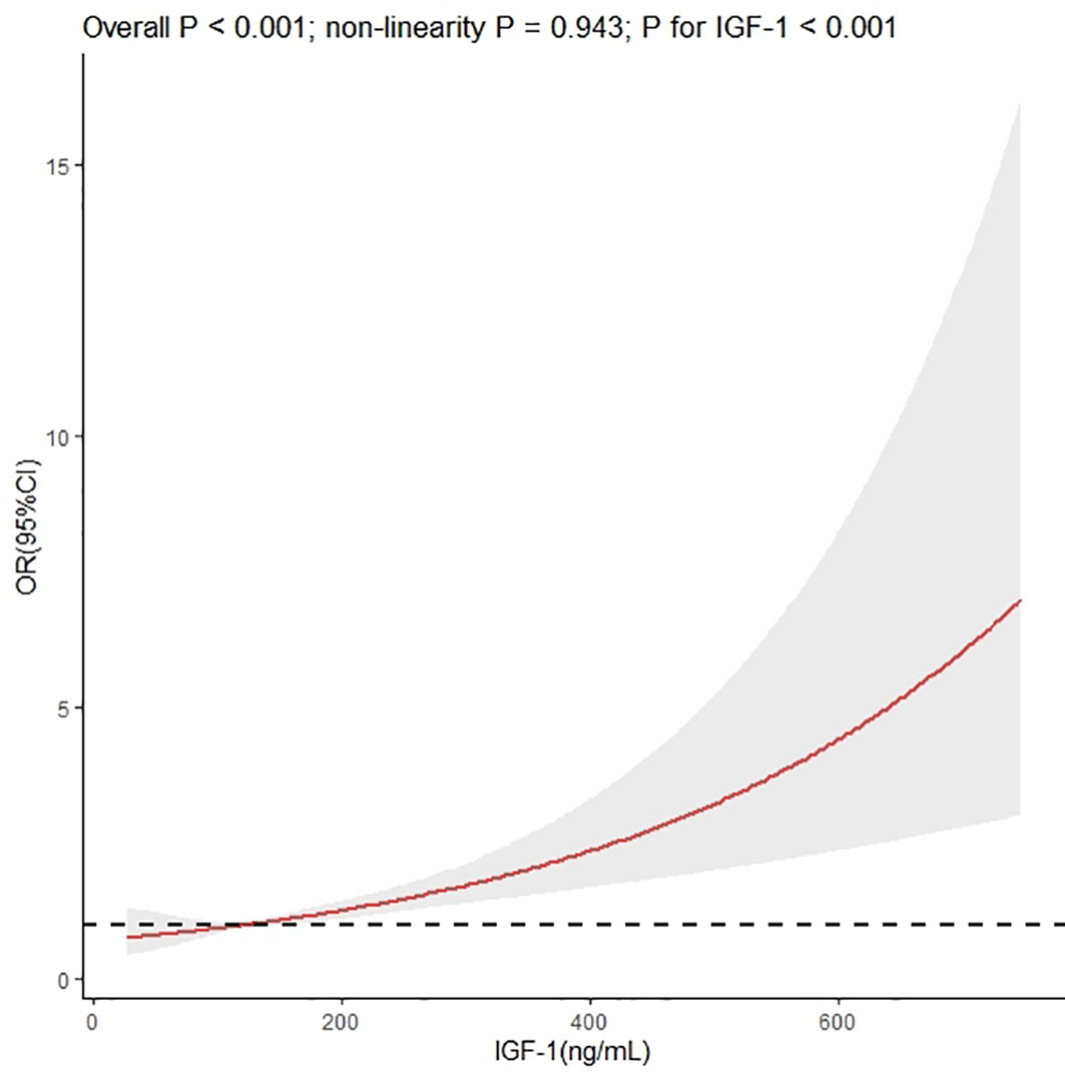
Dose–response association between insulin-like growth factor-1 and interventricular septal thickening according to restricted cubic splines. The *asterisk* means adjusted for propensity scores.

**Figure 4:**
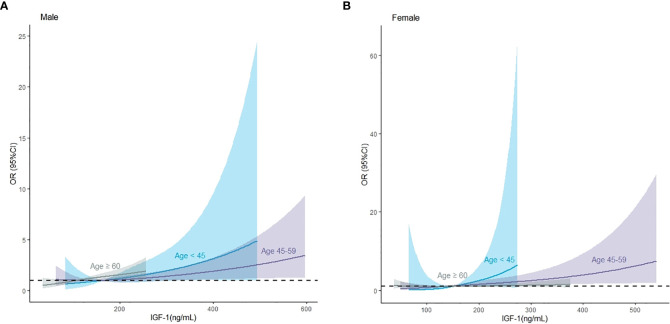
The association between insulin-like growth factor-1 and interventricular septal thickening according to restricted cubic splines in male and female patients, respectively. **(A)** Male population, **(B)** female population. The *asterisk* means adjusted for propensity scores.

We used restricted cubic splines to flexibly model and visualize the relationship between IGF-1 levels and the risk of IVS thickening. The multivariable-adjusted spline regression model showed a linear association between IGF-1 and the risk of IVS thickening (*P* for overall model <0.001, *P* for nonlinear = 0.943, and *P* for IGF-1 < 0.001) (**Figure 3**). In splines for the age and sex subgroups, different IGF-1 levels increased the risk of IVS thickening among different age groups in male patients: 18–44 years when IGF-1 value >164.00 ng/mL, 45–60 years when IGF-1 value >140.34 ng/mL, and ≥60 years when IGF-1 value >108.20 ng/mL (**Figure 4A**). In female patients aged 45–60 years, the risk of IVS thickening increased when the IGF-1 levels were >207.45 ng/mL. However, the IGF-1 levels were not significantly correlated with the risk of IVS thickening in female patients aged 18–45 and ≥60 years (**Figure 4B**).

### Stratified analysis

Subgroup analyses for IVS thickening based on baseline information were performed after adjusting for propensity scores. The main results did not change significantly in most subgroups. No interaction was found in most subgroups except for the presence of acromegaly (*P* = 0.047 for interaction) when IGF-1 was treated as a continuous variable. The associated risk of IVS thickening increased more markedly in patients without acromegaly (OR, 1.003; 95% CI, 1.001–1.005, *P* < 0.01) than in those with acromegaly (OR, 1.000; 95% CI, 0.997–1.003, *P* > 0.05) (**Table 3**).

**Table 3:** Association between insulin-like growth factor-1 and interventricular septal thickening in various subgroups.

Variables	Case/Number	Continuous, IGF–I	*P*-value	*P* for interaction	Categorical, T3 vs T1–2	*P*-value	*P* for interaction
		OR (95% CI)*			OR (95% CI)*		
Age							
18–44 years	21/105	1.006 (1.002–1.012)	0.010		3.775 (1.298–12.842)	0.021	
45–59 years	71/323	1.003 (1.002–1.005)	<0.001		1.706 (0.970–3.011)	0.064	
≥60 years	88/698	1.003 (1.001–1.005)	0.013	0.221	1.847 (1.035–3.252)	0.035	0.708
Sex							
Male	116/479	1.003 (1.001–1.005)	<0.001		1.454 (0.925–2.280)	0.103	
Female	64/324	1.003 (1.002–1.005)	<0.001	0.695	2.834 (1.545–5.229)	<0.001	0.099
Hypertension							
No	40/174	1.004 (1.002–1.007)	<0.001		1.596 (0.737–3.441)	0.231	
Yes	140/629	1.003 (1.001–1.004)	<0.001	0.181	1.896 (1.258–2.852)	0.002	0.770
DM							
No	92/410	1.003 (1.002–1.005)	<0.001		1.835 (1.114–3.019)	0.017	
Yes	88/393	1.003 (1.001–1.005)	<0.001	0.978	1.835 (1.084–3.095)	0.023	0.981
BMI							
<28	101/499	1.004(1.002–1.006)	<0.001		1.567 (0.959–2.545)	0.07	
≥28	79/304	1.002(1.001–1.004)	0.004	0.220	2.223 (1.293–3.843)	0.004	0.579
Acromegaly							
No	156/763	1.003 (1.001–1.005)	0.002		1.484 (0.997–2.197)	0.05	
Yes	24/40	1.000 (0.997–1.003)	0.872	0.047	–	0.985	0.981
CAD							
No	134/638	1.003 (1.002–1.004)	<0.001		1.635 (1.083–2.465)	0.019	
Yes	46/165	1.005 (1.001–1.010)	0.036	0.374	2.732 (1.265–5.942)	0.01	0.192
SBP (mmHg)							
<140	78/398	1.004 (1.002–1.006)	<0.001		1.817 (1.038–3.160)	0.035	
≥140	102/405	1.002 (1.001–1.004)	0.004	0.471	1.867 (1.158–3.013)	0.01	0.333
DBP (mmHg)							
<90	116/534	1.004 (1.002–1.005)	<0.001		1.675 (1.057–2.641)	0.027	
≥90	64/269	1.003 (1.001–1.005)	0.003	0.343	2.317 (1.255–4.340)	0.008	0.155

BMI, body mass index; CAD, coronary artery disease; DM, diabetes mellitus; SBP, systolic blood pressure; DBP, diastolic blood pressure.

* Adjusted for propensity scores.

Additionally, we stratified by sex, age, and presence of acromegaly. In female patients, elevated IGF-1 was associated with an increased risk of IVS thickening in the following groups: 18–44 years (OR, 1.018; 95% CI, 1.004–1.039; *P* < 0.05), 45–59 years (OR, 1.005; 95% CI, 1.002–1.009; *P* < 0.01), and absence of acromegaly (OR, 1.003; 95% CI, 1.001–1.006; *P* < 0.01). In male patients, elevated IGF-1 was associated with an increased risk of IVS thickening in the following groups: 18–44 years (OR, 1.005; 95% CI, 1.001–1.011; *P* < 0.05), 45–59 years (OR, 1.002; 95% CI, 1.001–1.005; *P* < 0.05), and ≥ 60 years (OR, 1.005; 95% CI, 1.001–1.009; *P* < 0.05). Nevertheless, in male patients, neither age nor the presence of acromegaly significantly modified the association (*P* for all interactions > 0.05), except for the presence of acromegaly (*P* for interaction = 0.026) in female patients (**Table 4**).

**Table 4:** Association between insulin-like growth factor-1 and interventricular septal thickening stratified by sex, age, and acromegaly.

Variables	Case/number	Continuous, IGF-1	*P*-value	*P* for interaction
		OR (95% CI)^a^		
Male				
18–44 years	13/65	1.005 (1.001–1.011)	0.045	
45–59 years	46/205	1.002 (1.001–1.005)	0.016	
≥60 years	57/209	1.005 (1.001–1.009)	0.013	0.089
Acromegaly (-)	116/456	1.003 (1.000–1.005)	0.050	
Acromegaly (+)	15/23	1.001 (0.997–1.005)	0.635	0.309
Female				
18–44 years	8/40	1.018 (1.004–1.039)	0.042	
45–59 years	25/118	1.005 (1.002–1.009)	0.002	
≥60 years	25/166	1.001 (0.998–1.004)	0.322	0.026
Acromegaly (–)	55/307	1.003 (1.001–1.006)	0.025	
Acromegaly (+)	9/17	0.998 (0.992–1.003)	0.513	0.127

IGF-1, insulin-like growth hormone-1; IVS thickening, interventricular septal thickening.

a Adjusted for propensity scores.

### Sensitivity analysis

Sensitivity analysis was performed after excluding 40 patients with acromegaly, and the results revealing the relationship between IGF-1 levels and the risk of IVS thickening, which was independent of acromegaly, were stable (**Table 2 in Supplement 2**).

## Discussion

Our results demonstrated that elevated IGF-1 was independently associated with an increased risk of IVS thickening. In addition, these results and trends were stable in both sensitivity analysis and subgroup analysis. This implies that the relationship between IGF-1 levels and the risk of IVS thickening is robust and reliable after excluding acromegaly.

Hypertension is known to be an important factor leading to IVS thickening and left ventricular hypertrophy (18). However, we matched the levels of SBP and DBP and the presence of hypertension between the case and control groups in our study. In addition, we adjusted for the presence of hypertension and levels of SBP and DBP as covariates. A study in young healthy pilots showed that IVS thickening may not merely be a result of long-term elevation in BP but might also predict future systolic hypertension (19). Another clinical study in a healthy population also found an association between IVS thickening and SBP and heart rate, but not DBP (6). Consequently, IVS thickening can be seen in healthy populations as well as in hypertensive populations. Therefore, it is necessary to study the risk factors associated with IVS in addition to hypertension. Our study revealed that plasma IGF-1 levels may be a risk factor for IVS thickening in patients with/without hypertension.

Among the multivariable analysis included in the logistic regression model, IGF-1 levels maintained an independent association with IVS thickening, regardless of other confounders. This relationship suggests that IGF-1 levels may be responsible for IVS thickening even in the presence of diabetes, hypertension, obesity, and other diseases known to affect the heart structure and function. Abnormal serum IGF-1 levels are associated with an increased risk of IVS thickening. Our study identified that IGF-1 levels were associated with the risk of IVS thickening regardless of underlying conditions. Additionally, we also found that the risk thresholds for the three age groups (18–44, 45–59, and ≥60 years) were different in male and female patients, respectively.

IGF-1 has many mitogenic effects, including stimulation of cell growth, division, and differentiation through specific receptors on target cell surfaces (20). It also has an anabolic effect, enhancing glucose and amino acid uptake and inhibiting protein breakdown (21, 22). IGF-1 is expressed throughout the post-natal period and in adulthood (23). In patients with acromegaly, sustained high levels of GH and IGF-1 stimulate the hearts and interacted with the GH and IGF-1 receptors, respectively, on the surface of cardiomyocytes (24). Myocardial contractility changes with an increase in intracellular calcium concentration and calcium sensitivity, resulting in collagen precipitation, muscle fiber disorder, and lymphocyte infiltration in the interstitial of cardiomyocytes, which eventually develops into acromegaly cardiomyopathy (25). Moreover, IGF-1 indirectly affects the cardiovascular system by increasing insulin sensitivity (26–28). Since insulin is a well-known growth factor, probably hyperinsulinemia and insulin resistance may contribute to IVS thickening. Evidence suggests that insulin levels and insulin resistance are associated with myocardial steatosis, cardiac remodeling, and fitness in women with obesity (29). IGF-1 regulates a number of cellular processes in the heart, including senescence, apoptosis, growth, metabolism, and autophagy (30, 31). Animal studies found that IGF-1 activates the PI3K/Akt signaling pathway and regulates cardiomyocyte survival, hypertrophy, and senescence (13–15). IGF-1 promotes angiogenesis and nascent vessel formation (32, 33) and stimulates cardiomyocyte growth, which may be associated with IVS thickening.

We performed sensitivity analysis by excluding patients with acromegaly, and the results showed that the relationship between IGF-1 levels and IVS thickening is independent of acromegaly. In addition, our stratified analysis also suggested that the relationship between IGF-1 and IVS thickening is derived from the patients without acromegaly. To the best of our knowledge, this is the first study to investigate the role of IGF-1 in IVS thickening after excluding patients with acromegaly. We found that IGF-1 levels are associated with the risk of IVS thickening in patients with or without diabetes, hypertension, and obesity in a stratified analysis. This result emphasizes that IGF-1 levels should be measured to assess the risk of IVS thickening and subsequent onset of cardiovascular complications.

Our study has some limitations. Firstly, because of the case–control setting, this study directly prompted that IGF-1 levels might be a risk for IVS thickening. Therefore, prospective studies are needed to clarify the causal relationship. Secondly, considering that isolated IVS thickening can be seen in patients with hypertension or DM, we did not analyze the interference with all possible medications. However, we found no statistically significant difference between the two groups for antihypertensive agents, including ACEI/ARB, and adjusted for the covariates. Thirdly, though our patients were from a single center, the patients were from multiple cities. Fourthly, IGF-1 is not a routine examination item, and not all patients have these relevant data, creating a potential selection bias. Finally, as we could not control all possible confounding factors, we did not measure and collect some factors. However, during analysis, we performed propensity score matching and adjusting, which may be a better statistical method to control confounding factors.

## Conclusion

Our study showed that plasma IGF-1 levels are associated with the risk of IVS thickening. More prospective studies and animal studies are needed to explore the relationship between IGF-1 and IVS thickening as well as underlying mechanisms.

## Data availability statement

The raw data supporting the conclusions of this article will be made available by the authors without undue reservation.

## Ethics statement 

The studies involving human participants were reviewed and approved by the Ethics Committee of People’s Hospital of Xinjiang Uygur Autonomous Region. Written informed consent for participation was not required for this study in accordance with the national legislation and the institutional requirements.

## Author contributions

YC, XC carried out the statistical analysis and prepared the original manuscript. YC, XC and YG contributed to conceive and design the present study. SL, YY and SX collected the data.
