# Supplementary material for: Insulin-like growth factor-1 levels are associated with interventricular septal thickening
Source: Front Endocrinol (Lausanne). 2022 Dec 7;13:997023. doi: 10.3389/fendo.2022.997023 (PMC9768022; doi:10.3389/fendo.2022.997023)
Supplement: Supplementary file 1 [file Table_1.docx]

**TABLE supplement 1 The result of collinearity statistics**

|  | Tolerance | VIF | Tolerance | VIF |
| --- | --- | --- | --- | --- |
| Age | 0.56 | 1.785 | 0.564 | 1.774 |
| Sex | 0.547 | 1.829 | 0.551 | 1.816 |
| Smoking | 0.476 | 2.1 | 0.481 | 2.081 |
| Alcohol consumption status | 0.549 | 1.82 | 0.55 | 1.819 |
| SBP | 0.46 | 2.175 | 0.461 | 2.17 |
| DBP | 0.397 | 2.52 | 0.4 | 2.501 |
| BMI | 0.729 | 1.372 | 0.734 | 1.362 |
| Heart rate | 0.777 | 1.286 | 0.783 | 1.276 |
| ALT | 0.014 | 69.344 | 0.698 | 1.433 |
| AST | 0.015 | 68.006 | eliminate | eliminate |
| γ-GGT | 0.662 | 1.511 | 0.729 | 1.371 |
| ALP | 0.756 | 1.324 | 0.76 | 1.316 |
| Albumin | 0.728 | 1.374 | 0.739 | 1.354 |
| Cr | 0.648 | 1.542 | 0.65 | 1.539 |
| BUN | 0.831 | 1.204 | 0.832 | 1.202 |
| UA | 0.626 | 1.597 | 0.631 | 1.586 |
| TG | 0.271 | 3.684 | 0.826 | 1.21 |
| LDL | 0.165 | 6.078 | 0.802 | 1.247 |
| TC | 0.1 | 10.009 | eliminate | eliminate |
| HDL | 0.513 | 1.949 | 0.808 | 1.238 |
| Serum calcium | 0.645 | 1.552 | 0.645 | 1.549 |
| Serum potassium | 0.689 | 1.451 | 0.69 | 1.449 |
| Serum natrium | 0.799 | 1.251 | 0.803 | 1.245 |
| Serum phosphate | 0.974 | 1.027 | 0.975 | 1.026 |
| FBG | 0.68 | 1.47 | 0.685 | 1.461 |
| CK | 0.749 | 1.334 | 0.75 | 1.333 |
| CK-MB | 0.749 | 1.335 | 0.755 | 1.325 |
| WBC | 0.696 | 1.437 | 0.698 | 1.432 |
| PLT | 0.722 | 1.385 | 0.726 | 1.377 |
| Hb | 0.546 | 1.831 | 0.554 | 1.804 |
| D-Dimer | 0.581 | 1.722 | 0.583 | 1.714 |
| Cortisol | 0.754 | 1.325 | 0.759 | 1.318 |
| ACTH | 0.918 | 1.089 | 0.92 | 1.087 |
| TSH | 0.889 | 1.125 | 0.893 | 1.119 |
| FT3 | 0.164 | 6.082 | 0.447 | 2.239 |
| FT4 | 0.233 | 4.3 | 0.341 | 2.935 |
| TT3 | 0.149 | 6.691 | eliminate | eliminate |
| TT4 | 0.185 | 5.398 | 0.334 | 2.991 |
| Diabetes mellitus | 0.763 | 1.31 | 0.767 | 1.303 |
| Hypertension | 0.719 | 1.392 | 0.72 | 1.39 |
| CAD | 0.68 | 1.471 | 0.687 | 1.455 |
| GH | 0.653 | 1.532 | 0.653 | 1.531 |
| IGF-I | 0.577 | 1.732 | 0.579 | 1.726 |
| ACEI/ARB | 0.779 | 1.284 | 0.785 | 1.274 |
| βblockers | 0.824 | 1.213 | 0.843 | 1.186 |
| Calcium channel blockers | 0.778 | 1.285 | 0.779 | 1.284 |
| Diuretics | 0.788 | 1.269 | 0.799 | 1.252 |
| Antiplatelet | 0.562 | 1.78 | 0.567 | 1.763 |
| Statins | 0.572 | 1.748 | 0.582 | 1.717 |

SBP: systolic blood pressure, DBP: diastolic blood pressure, BMI: body mass index, CAD: coronary artery disease, DM: diabetes mellitus, ALT: alanine transaminase, AST: glutamic oxalacetic transaminase,γ-GGT: γ-gamma-glutamyltransferase, ALP: alkaline phosphatase, Cr: creatinine, BUN: blood urea nitrogen, UA: uric acid,TG: triglyceride, TC: total cholesterol, LDL–C: low-density lipoprotein–cholesterol, HDL–C: high-density lipoprotein–cholesterol, FBG: fasting blood glucose, CK: creatine kinase, CK-MB: creatine kinase isoenzyme MB, WBC: white blood cell, PLT: plastocyte, Hb: hemoglobin, ACTH: adrenalotropic hormone, TSH: thyroid stimulating hormone, FT3: free Triiodothyronine, FT4: free Thyroxine, TT3: total Triiodothyronine, TT4: total Thyroxine, IGF-1: Insulin-Like Growth Hormone-1, GH: Growth Hormone and ACE/ARB: ACEI/ARB: angiotensin-converting enzyme/angiotensin 2 receptor inhibitors.
