# Supplementary material for: Insulin-like growth factor-1 levels are associated with interventricular septal thickening
Source: Front Endocrinol (Lausanne). 2022 Dec 7;13:997023. doi: 10.3389/fendo.2022.997023 (PMC9768022; doi:10.3389/fendo.2022.997023)
Supplement: Supplementary file 2 [file Table_2.docx]

**TABLE supplement 2 The association between the insulin-like growth factor-1 and interventricular septal thickening excluding patients with acromegaly.**

|  | **Univariate Model** | | | **Model 1** | | | **Model 2** | | |
| --- | --- | --- | --- | --- | --- | --- | --- | --- | --- |
|  | **OR** | **95%CI** | **P-value** | **OR** | **95%CI** | **P-value** | **OR** | **95%CI** | **P-value** |
| IGF-1 per 1 (ng/mL) | 1.003 | 1.001-1.005 | 0.003 | 1.004 | 1.002-1.006 | <0.001 | 1.003 | 1.001-1.005 | 0.003 |
| IGF-1 per 5 (ng/mL) | 1.014 | 1.005-1.024 | 0.003 | 1.018 | 1.008-1.028 | <0.001 | 1.015 | 1.005-1.026 | 0.003 |
| IGF-1 per 10 (ng/mL) | 1.029 | 1.010-1.049 | 0.003 | 1.036 | 1.016-1.057 | <0.001 | 1.031 | 1.011-1.052 | 0.003 |

Mode 1: adjusting for age, sex, BMI, DBP, SBP, hypertension, T2DM, CAD.

Mode 2: adjusting for propensity scores.

IGF-1: Insulin-Like Growth Hormone-1 and IVS thickening: interventricular septal thickening.
